# Palmitic Acid Methyl Ester Enhances Adipogenic Differentiation in Rat Adipose Tissue-Derived Mesenchymal Stem Cells through a G Protein-Coupled Receptor-Mediated Pathway

**DOI:** 10.1155/2021/9938649

**Published:** 2021-10-05

**Authors:** Jian-Hong Lin, Huan-Hsin Chang, Wen-Sen Lee, Pei-Ching Ting, Yu-Po Luo, Kun-Ta Yang

**Affiliations:** ^1^PhD Program in Pharmacology and Toxicology, School of Medicine, Tzu Chi University, No. 701, Sec. 3, Zhongyang Rd., Hualien, Taiwan; ^2^Division of Experimental Surgery, Department of Surgery, Hualien Tzu Chi Hospital, Buddhist Tzu Chi Medical Foundation, No. 707, Sec. 3, Zhongyang Rd., Hualien, Taiwan; ^3^Master Program in Medical Physiology, School of Medicine, Tzu Chi University, No. 701, Sec. 3, Zhongyang Rd., Hualien, Taiwan; ^4^Graduate Institute of Medical Sciences, College of Medicine, Taipei Medical University, No. 250, Wuxing St., Taipei, Taiwan; ^5^Department of Surgery, Hualien Tzu Chi Hospital, Buddhist Tzu Chi Medical Foundation, No. 707, Sec. 3, Zhongyang Rd., Hualien, Taiwan; ^6^Department of Physiology, School of Medicine, Tzu Chi University, No. 701, Sec. 3, Zhongyang Rd., Hualien, Taiwan

## Abstract

Adipogenic differentiation from stem cells has become a research target due to the increasing interest in obesity. It has been indicated that adipocytes can secrete palmitic acid methyl ester (PAME), which is able to regulate stem cell proliferation. However, the effects of PAME on adipogenic differentiation in stem cell remain unclear. Here, we present that the adipogenic differentiation medium supplemented with PAME induced the differentiation of rat adipose tissue-derived mesenchymal stem cells (rAD-MSCs) into adipocyte. rAD-MSCs were treated with PAME for 12 days and then subjected to various analyses. The results from the present study show that PAME significantly increased the levels of adipogenic differentiation markers, PPAR*γ* and *Gpd1*, and enhanced adipogenic differentiation in rAD-MSCs. Furthermore, the level of GPR40/120 protein increased during induction of adipocyte differentiation in rAD-MSCs. Cotreatment with PAME and a GPR40/120 antagonist together inhibited the PAME-enhanced adipogenic differentiation. Moreover, PAME significantly increased phosphorylation of extracellular signal-regulated kinases (ERK), but not AKT and mTOR. Cotreatment with PAME and a GPR40/120 antagonist together inhibited the PAME-enhanced ERK phosphorylation and adipogenic differentiation. PAME also increased the intracellular Ca^2+^ levels. Cotreatment with PAME and a Ca^2+^ chelator or a phospholipase C (PLC) inhibitor prevented the PAME-enhanced ERK phosphorylation and adipogenic differentiation. Our data suggest that PAME activated the GPR40/120/PLC-mediated pathway, which in turn increased the intracellular Ca^2+^ levels, thereby activating the ERK, and eventually enhanced adipogenic differentiation in rAD-MSCs. The findings from the present study might help get insight into the physiological roles and molecular mechanism of PAME in regulating stem cell differentiation.

## 1. Introduction

Obesity is a tremendous health problem worldwide. Excess adiposity is an established risk factor for metabolic diseases, heart diseases, hypertension, stroke, and several types of cancer [1]. Obesity is defined as an excessive accumulation of adipose tissue and can be divided into two main types: hyperplasia (adipocyte number increase) and hypertrophy (adipocyte size increase). However, several studies have demonstrated that the adipocyte number increases when body fat reaches 25% of the total body weight in children and adults [2, 3]. Interestingly, adipocyte precursors from obese subjects proliferate more rapidly in culture than the cells from lean individuals [4, 5]. New adipocytes constantly arise from a preexisting population of undifferentiated progenitor cells. On the other hand, the mesenchymal stem cells (MSCs), a major source of adipocyte generation in adipose tissue, can increase adipocyte number [1, 6].

Adipose tissue is now recognized as an endocrine organ that secretes numerous adipokines and free fatty acids (FFAs). Previous studies have shown that adipose tissue environmental niche, in which adipose-derived mesenchymal stem cells (AD-MSCs) reside, has a profound impact on their differentiation capacity [7–9]. The adipocytes can secrete factors, such as sFRP-1 [10], chemerin [11], adiponectin [12], and leptin [13], to promote adipogenic differentiation. Similarly, the adipose tissue condition medium increases adipogenic differentiation of AD-MSCs. On the other hand, FFAs, such as palmitate [14], arachidonic acid [15], and linoelaidic acid [16], have been reported to enhance adipogenic differentiation in MSCs.

A recent study reported that perivascular adipose tissue releases PAME, causing vasorelaxation [17]. PAME, an endogenous fatty acid methyl ester (FAME), has been reported to possess potent antifibrotic [18–20] and anti-inflammatory activities [21, 22] and regulates cell proliferation in MSCs [23–25]. PAME is detected in adipogenic differentiated mesenchymal stromal cells, but not in the undifferentiated mesenchymal stromal cells [26]. Taken together, these data suggest that PAME plays a critical role in the stem cell differentiation. However, the effects of PAME on the differentiation of stem cell into the adipocyte lineage remain unclear.

PAME has a structure similar to fatty acid, but its target molecule is still unclear. Several orphan G protein-coupled receptors (GPCRs), including GPR40, GPR43, GPR41, and GPR120, have been identified as receptors for free fatty acid and can be activated by free fatty acid and their derivatives. GPR43 and GPR41 are activated by short-chain fatty acids, whereas GPR40 and GPR120 are activated by medium- to long-chain fatty acids [27].

Ichimura et al. demonstrated in a human study that the level of GPR120 is significantly higher in obese individuals than in lean control [28]. Furthermore, the level of GPR120 mRNA increases during induction of adipocyte differentiation in 3T3-L1 cells [29]. Knockdown of GPR120 by siRNA or GPR120 gene deficiency suppresses the expression of adipogenic genes and lipid accumulation in 3T3-L1 cells and mouse embryonic fibroblast, respectively [28, 29]. Gao et al. also demonstrated that low concentrations of GPR120 agonists can increase the ability of adipogenic differentiation in MSCs [30]. However, the mechanism underlying PAME enhancing the GPR40/120-mediated adipocyte differentiation in rat AD-MSCs (rAD-MSCs) has not been elucidated. In the present investigation, we delineated the signaling pathway involved in the PAME-enhanced rAD-MSC adipogenic differentiation.

## 2. Materials and Methods

### 2.1. Chemicals

PAME, palmitic acid (PA), and stearic acid methyl ester (SAME) were purchased from Sigma-Aldrich (St. Louis, MO) and dissolved in 100% methanol. DC260126, dexamethasone, L-ascorbate-2-phosphate, indomethacin, 1,2-bis (2-aminophenoxy)ethane-N,N,N′,N′-tetraacetic acid tetra (acetoxymethyl ester) (BAPTA-AM), triethyl phosphate, and Oil Red O were purchased from Sigma-Aldrich. GW1100 was purchased from Cayman Chemical Company (Ann Arbor, MI), AH7614 was purchased from Abcam (Cambridge, England), U73122 was purchased from Millipore (Burlington, MA), and U0126 was purchased from Calbiochem (San Diego, CA).

### 2.2. Preparation and Expansion of rAD-MSCs

Adult male Sprague-Dawley rats (350-450 g) were used as adipose tissue donors. The rAD-MSCs were obtained from the adipose tissues of rats that were anesthetized using urethane (1.5 g/kg, i.p.) and sacrificed. The protocol was approved by the Institution of Animal Care and Use Committee of the Tzu Chi University (IACUC Approval No. 107003). Adipose tissues were washed using phosphate-buffered saline (PBS) containing 1% penicillin/streptomycin (GIBCO; Grand Island, NY). The tissues were minced and digested with 0.1% collagenase type I (GIBCO) in PBS for 60 min at 37°C, and collagenase was then inactivated with an equal amount of culture medium (DMEM-high glucose supplemented with 10% FBS and 1% penicillin/streptomycin) (GIBCO). After centrifugation for 10 min at 2,000 rpm, the rAD-MSCs were incubated at 37°C in a fully humidified atmosphere with 5% CO_2_, and the culture medium was changed every 48 h.

### 2.3. Adipogenic Differentiation

rAD-MSCs were seeded at a density of 1 × 10^4^ cells/cm^2^. Cells were incubated in a humidified incubator at 37°C with 5% CO_2_. Adipocyte differentiation was induced by replacing culture medium with adipogenic induction medium (DMEM-high glucose supplemented with 10^−7^ M dexamethasone, 50 *μ*g/mL L-ascorbate-2-phosphate, 10 *μ*g/mL indomethacin, 10% FBS, and 1% penicillin/streptomycin). The adipogenic induction medium was changed every other day for 12 days.

### 2.4. Oil Red O Staining

rAD-MSCs were seeded in 24 mm poly-l-lysine-coated coverslips. After differentiation into adipocytes, adipogenic induction medium was removed and the cells were washed thrice with PBS. Cells were fixed in 10% formaldehyde for 30 min at room temperature and washed thrice with distilled water. Distilled water was then removed from the coverslips, and 500 *μ*L of Oil Red O working solution (from 3 mL of stock solution, which contains 250 mg Oil Red O in 50 mL 60% triethyl phosphate, in 2 mL distilled water, and filtered through Whatman filter paper No. 1.) was added to each coverslip. Cells were stained for 10 min at room temperature and then washed thrice with distilled water. Cells were examined under the microscope, and images were captured at 10x magnifications using an Olympus CX31 microscope. For quantitation of lipid droplets, cells were extracted with 200 *μ*L of 60% triethyl phosphate for 10 min, and absorbance was measured using Thermo Scientific Multiskan Spectrum ELISA plate reader (Thermo, Waltham, MA) at 490 nm.

### 2.5. Western Blot Analysis

rAD-MSCs were washed twice with PBS and lysed in RIPA lysis buffer (Millipore) containing 1% protease inhibitor (Calbiochem) and 0.5% phosphatase inhibitor (Calbiochem). The lysates were clarified by centrifugation at 12,000 rpm for 15 min at 4°C. Electrophoresis sample buffer (1 M Tris–HCl, pH 6.8; 5% 2-mercaptoethanol; 20% glycerol; 10% SDS; and 0.5% bromophenol blue) was added to the cell lysates and boiled for 10 min at 110°C. The protein sample (30 *μ*g) was loaded into a 10% SDS-polyacrylamide gel, subjected to electrophoresis, and then transferred to PVDF membrane (Millipore). The membrane was treated with 5% BSA in Tween-Tris-buffered saline (T-TBS) buffer (0.05% Tween 20; 200 mM Tris-HCl, pH 7.4; and 1.5 M NaCl) for 1 h at room temperature to block the nonspecific IgGs and then incubated with primary antibodies diluted in T-TBS buffer overnight at 4°C. The information of primary and secondary antibodies used in this study is shown as follows: all primary antibodies including anti-PPAR*γ*, anti-p-ERK1/2, anti-ERK1/2, anti-p-AKT, anti-AKT, anti-p-mTOR, and anti-mTOR purchased from Cell Signaling (Danvers, MA); anti-GPR40 purchased from Boster Bio (Pleasanton, CA); and anti-GPR120 purchased from Novus Biologicals (Centennial, CO) were in 1,000 dilution; anti-*β*-actin purchased from Millipore was in 10,000 dilution. Secondary antibodies (peroxidase-conjugated goat anti-rabbit IgG or anti-mouse IgG) were diluted in T-TBS buffer (1 : 5,000), and the immunoreactive proteins were visualized with an ECL detection system (GE Healthcare; Uppsala, Sweden). The resulting bands were analyzed using Image-Pro Plus software and normalized using *β*-actin.

### 2.6. Quantitative Real-Time PCR

Total RNAs were extracted from rAD-MSCs using the Trizol reagent (Ambion; Carlsbad, CA), and the cDNA was synthesized with Verso™ cDNA Kit (Thermo) using 3 *μ*g of total RNAs. The quantitation of cDNA amount was performed with quantitative real time-PCR using a Maxima SYBR Green qPCR Master Mix (2x) and ROX Solution (Thermo) and then detected using the LightCycler 480 System (Roche; Basel, Switzerland). The following PCR primers were used: *Pparγ*, forward: 5′-TTG AGT GCC GAG TCT GTG GGG ATA A-3′, reverse: 5′-CAG GGA GGC CAG CAT CGT GTA GA-3′; *Gpd1*, forward: 5′-AAC AAT GAC CAC GAA AAC GTG A-3′, reverse: 5′-GTG AGG GAT GAC GAA CAC CA-3′; *Gapdh*, forward: 5′- ATG TTC CAG TAT GAC TCC ACT CAC G-3′, reverse: 5′-GAA GAC ACC AGT AGA CTC CAC GAC A-3′. All gene expression was analyzed using the comparative *C*_t_ method (2^-*ΔΔ*Ct^), where ΔΔ*C*_t_ = Δ*C*_t_ (sample) − Δ*C*_t_ (reference) relative to *Gapdh* levels.

### 2.7. Flow Cytometry

rAD-MSCs were incubated with N-2-hydroxy-ethylpiperazine-N′-2-ethanesulphonic acid- (HEPES-) buffered Tyrode solution (NT) containing 2.5 *μ*M Fluo-3-AM (Invitrogen; Carlsbad, CA. USA) for 30 min at room temperature. Intracellular Ca^2+^ analyses were carried out using Gallios™ Flow Cytometer (Beckman Coulter; Brea, CA. USA). The excitation/emission was detected at 488/525 nm wavelength.

### 2.8. Statistical Analyses

Data from at least three independent experiments were presented as means ± SEM and compared with unpaired *t*-test. Data obtained from three or more groups were subjected to one-way ANOVA followed by Fisher's least significant difference test, and statistical value of ^∗^*p* < 0.05 was considered significant.

## 3. Results

### 3.1. Effects of PAME on rAD-MSC Adipogenic Differentiation

**Figure 1:**
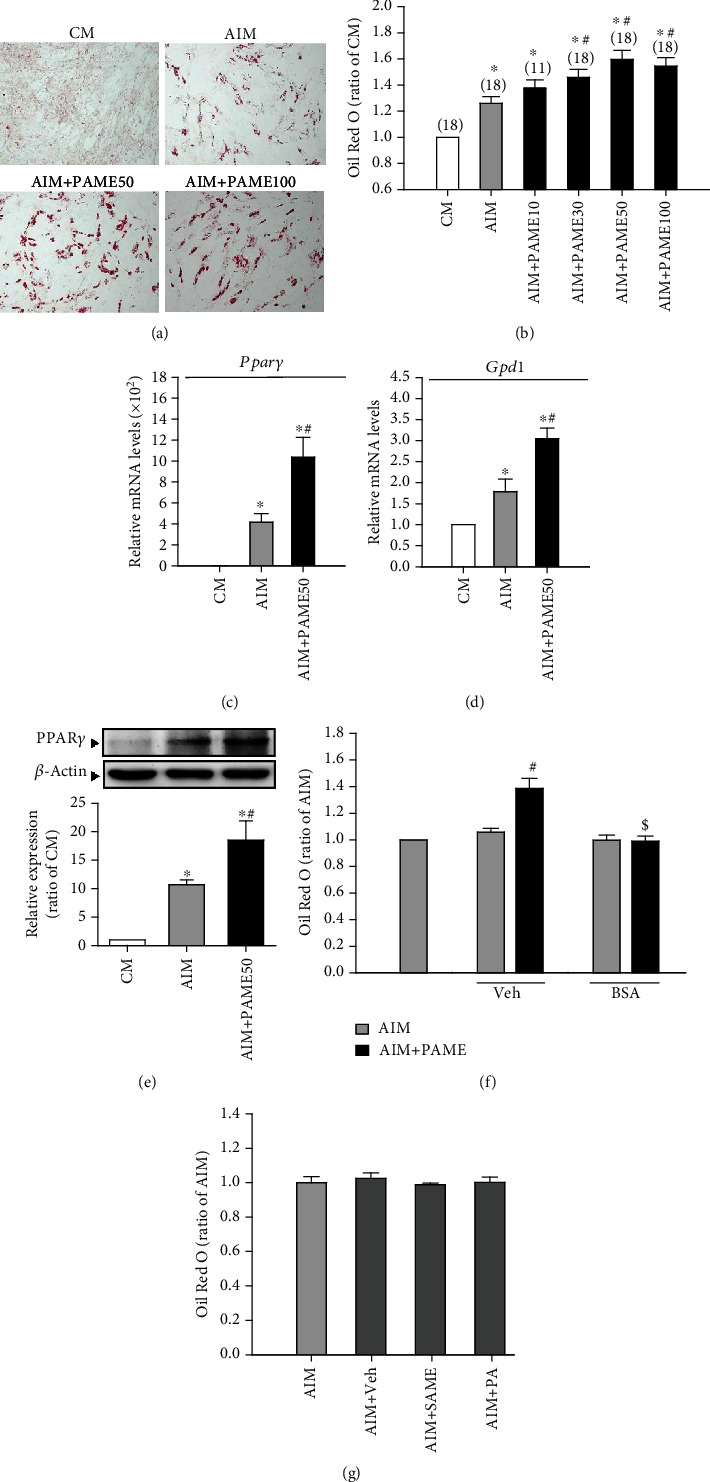
PAME increases adipogenic differentiation in rAD-MSCs. Treatment with PAME (10-100 *μ*M) in adipogenic induction medium for 12 days enhanced rAD-MSC adipogenic differentiation evidenced by Oil Red O staining (a) and quantitation of lipid droplets (b). Values shown in parentheses represent the number in each group. PAME (50 *μ*M) significantly increased the mRNA levels of *Pparγ* (c) (*n* = 10) and *Gpd1* (d) (*n* = 11) and the protein level of PPAR*γ* (e) (*n* = 5). (f) The effect of PAME on adipogenic differentiation was blocked by 0.5% BSA (*n* = 5). (g) Veh (0.1% methanol), SAME (50 *μ*M), and PA (50 *μ*M) did not enhance adipogenic differentiation of rAD-MSCs (*n* = 10). All data represent mean ± SEM. ^∗^*p* < 0.05, versus the CM group; ^#^*p* < 0.05, versus the AIM group; ^$^*p* < 0.05, versus the AIM+PAME group. CM: culture medium; AIM: adipogenic induction medium; BSA: bovine serum albumin; Veh: vehicle.

Initially, we examined the effect of PAME on rAD-MSC adipogenic differentiation. Treatment with PAME (30, 50, or 100 *μ*M) significantly enhanced rAD-MSC adipogenic differentiation (Figures 1(a) and 1(b)). The enhancement of PAME on the rAD-MSC adipogenic differentiation was confirmed by examining the mRNA and protein levels of adipogenic differentiation markers, peroxisome proliferator-activated receptor gamma (*Pparγ*), and glycerol-3-phosphate dehydrogenase 1 (*Gpd1*), in rAD-MSCs treated with or without PAME (50 *μ*M). Quantitative RT-PCR assay demonstrated that the mRNA levels of both *Pparγ* (Figure 1(c)) and *Gpd1* (Figure 1(d)) were significantly increased in the PAME-treated group as compared with the adipogenic induction medium-treated control group. The protein levels of PPAR*γ* detected by Western blot analyses also increased in the PAME-treated group (Figure 1(e)). PAME (50 *μ*M) significantly enhanced rAD-MSC adipogenic differentiation, and this enhancement was blocked by 0.5% BSA (Figure 1(f)). To further examine the specificity of PAME on rAD-MSC adipogenic differentiation, the adipogenic differentiation effect of SAME, a structural analog of FAME, and PA, a precursor of PAME, was tested to serve as negative controls. As shown in (Figure 1(g)), treatment with SAME (50 *μ*M) or PA (50 *μ*M) for 12 days did not significantly affect the adipogenic differentiation of rAD-MSCs.

### 3.2. Involvement of GPR40 and GPR120 in the PAME-Induced rAD-MSC Adipogenic Differentiation

**Figure 2:**
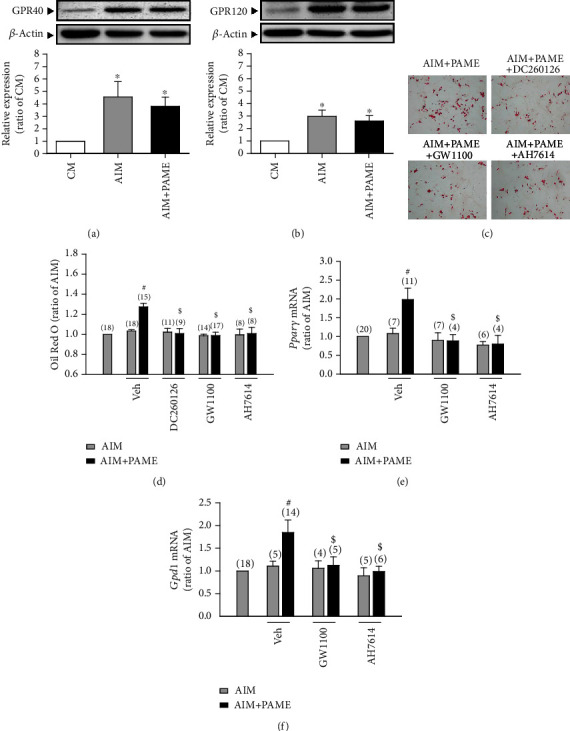
PAME activates the ERK1/2 pathway through stimulating the GPR40/120 in rAD-MSC adipogenic differentiation. Treatment with or without PAME (50 *μ*M) in adipogenic induction medium for 12 days enhanced the protein levels of GPR40 (a) and GPR120 (b) (*n* = 8). PAME (50 *μ*M) significantly enhanced the adipogenic differentiation, and this effect was inhibited by cotreatment with 5 *μ*M of GPR40 antagonist (DC260126 or GW1100) or 5 *μ*M of GPR120 antagonist (AH7614) (c, d). These antagonists were dissolved in 0.1% DMSO (vehicle). PAME significantly increased the mRNA levels of *Pparγ* (e) and *Gpd1* (f), and these effects were abolished by cotreatment with GW1100 or AH7614. Values shown in parentheses represent the number in each group. All data represent mean ± SEM. ^∗^*p* < 0.05, versus the CM group; ^#^*p* < 0.05, versus the AIM group; ^$^*p* < 0.05, versus the AIM+PAME group. CM: culture medium; AIM: adipogenic induction medium; Veh: vehicle.

The G protein-coupled receptors GPR40 and GPR120, also known as FFAR1 and FFAR4, respectively, are known to be activated by medium- to long-chain fatty acids [27]. Moreover, GPR40 has been shown to be the sensor of C10 methyl ester [31]. Accordingly, we examined whether GPR40/120 are involved in the PAME-enhanced rAD-MSC adipogenic differentiation. rAD-MSCs were induced to differentiate into adipocytes under the adipogenic induction medium. After induction of adipogenic differentiation with adipogenic induction medium for 12 days, the protein levels of both GPR40 (Figure 2(a)) and GPR120 (Figure 2(b)) were significantly increased in rAD-MSCs treated with or without PAME (50 *μ*M) group as compared with the cells treated with culture medium. Lipid droplets were stained with Oil Red O, and quantification of the staining was performed. As shown in Figures 2(c) and 2(d), treatment with PAME (50 *μ*M) enhanced the rAD-MSC adipogenic differentiation, and this effect was abolished by cotreatment with a GPR40 antagonist (DC260126 or GW1100) or a GPR120 antagonist (AH7614). To confirm the enhancement effect of PAME on the rAD-MSC adipogenic differentiation, the mRNA levels of the adipogenic differentiation markers, *Pparγ* and *Gpd1*, in rAD-MSCs were examined. Quantitative RT-PCR assay demonstrated that the mRNA levels of *Pparγ* (Figure 2(e)) and *Gpd1* (Figure 2(f)) were significantly decreased in the cells cotreated with PAME (50 *μ*M) and GW1100 or AH7614 as compared with the PAME-treated group.

### 3.3. Involvement of the PLC/ERK-Mediated Pathway in the PAME-Enhanced rAD-MSC Adipogenic Differentiation

**Figure 3:**
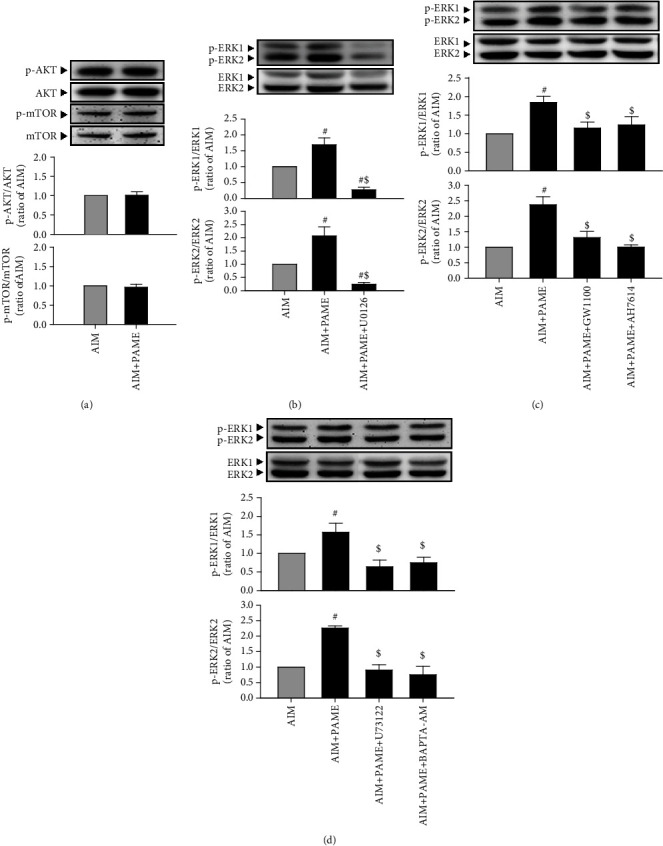
PAME activates the PLC-mediated signaling pathway through stimulating the GPR40/120 in rAD-MSCs. Treatment with PAME (50 *μ*M) in adipogenic induction medium for 12 days did not activate AKT and mTOR (a) (*n* = 12) but induced ERK1/2 activation (b) (*n* = 6). The PAME-induced ERK1/2 activation was abolished by cotreatment with ERK1/2 inhibitor (U0126). PAME (50 *μ*M) increased the levels of p-ERK1/2, and these effects were abolished by cotreatment with GW1100, AH7614 (c) (*n* = 4), U73122, or BAPTA-AM (d) (*n* = 4). All data represent mean ± SEM. ^#^*p* < 0.05, versus the AIM group; ^$^*p* < 0.05, versus the AIM+PAME group. AIM: adipogenic induction medium.

**Figure 4:**
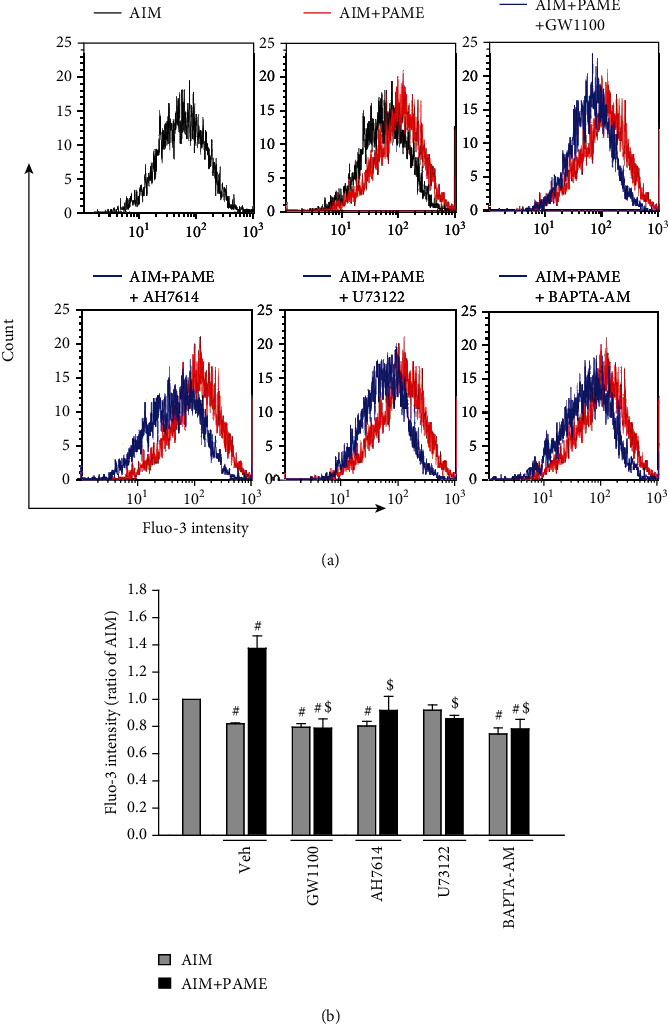
PAME increases intracellular Ca^2+^ through stimulating the GPR40/120 and activating the PLC-mediated signaling pathway in rAD-MSCs. Treatment with PAME (50 *μ*M) in adipogenic induction medium for 12 days increased the intracellular Ca^2+^ in rAD-MSCs, and these effects were abolished by cotreatment with GW1100, AH7614, U73122, or BAPTA-AM. (a) Representative histograms show the distribution of Fluo-3 fluorescence intensities, an indicator of intracellular Ca^2+^. (b) Quantitation fluorescence intensity of intracellular Ca^2+^ levels. All data represent mean ± SEM (*n* = 5). These antagonists were dissolved in 0.1% DMSO (vehicle). ^#^*p* < 0.05, versus the AIM group; ^$^*p* < 0.05, versus the AIM+PAME group. AIM: adipogenic induction medium; Veh: vehicle.

**Figure 5:**
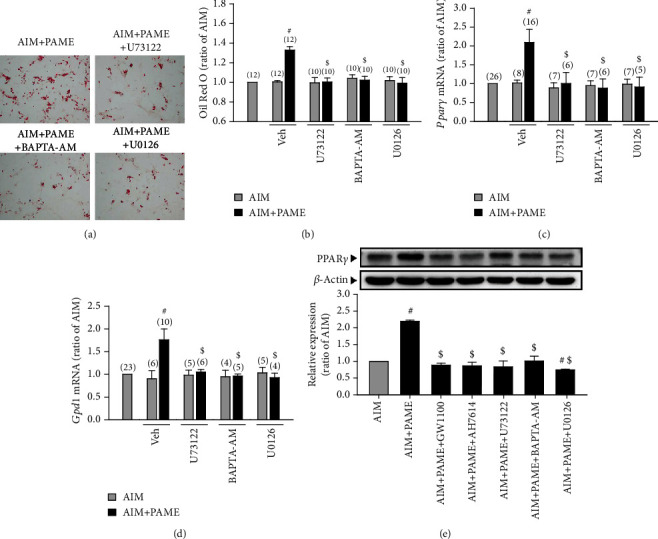
PAME increases adipogenic differentiation through activating the GPR40/120/PLC/ERK pathway. Treatment of rAD-MSCs with PAME (50 *μ*M) in adipogenic induction medium for 12 days enhanced the adipogenic differentiation, and these effects were abolished by cotreatment with U73122, U0126, or BAPTA-AM (a, b). PAME (50 *μ*M) significantly increased the mRNA levels of *Pparγ* (c) and *Gpd1* (d) in rAD-MSCs, and these effects were abolished by cotreatment with U73122, BAPTA-AM, or U0126. These antagonists were dissolved in 0.1% DMSO (vehicle). Values shown in parentheses represent the number in each group. PAME (50 *μ*M) increased the protein levels of PPAR*γ* in rAD-MSCs, and these effects were abolished by cotreatment with GW1100, AH7614, U73122, BAPTA-AM, or U0126 (e) (*n* = 3). All data represent mean ± SEM. ^#^*p* < 0.05, versus the AIM group; ^$^*p* < 0.05, versus the AIM+PAME group. AIM: adipogenic induction medium; Veh: vehicle.

Since GPR40 and GPR120 were coupled with Gq protein, subsequently activating the PI3K-AKT or ERK1/2 signaling pathway, which has been shown to be involved in stem cell differentiation [30, 32], and mTOR is a downstream protein of PI3K-AKT signaling pathway, it has been demonstrated that mTOR upregulates adipogenic transcriptional factors [33]; we further investigated whether the PI3K-AKT-mTOR or ERK1/2 signaling pathway is involved in the PAME-enhanced rAD-MSC adipogenic differentiation. Western blot analyses demonstrated that the level of p-ERK1/2 (Figure 3(b)), but not p-AKT and p-mTOR (Figure 3(a)), was increased by PAME (50 *μ*M) treatment. Cotreatment with PAME (50 *μ*M) and an ERK inhibitor, U0126 (5 *μ*M), abolished the PAME-enhanced phosphorylation of ERK1/2, suggesting that the PAME-enhanced rAD-MSC adipogenic differentiation might be mediated through activating the ERK1/2 signaling pathway. GPR40 and GPR120 were originally reported as receptors coupled with Gq protein [27] that activates phospholipase C (PLC), resulting in increased intracellular Ca^2+^ levels by inositol 1,4,5-trisphosphate- (IP_3_-) or diacylglycerol-induced phosphorylation of protein kinase C (PKC). Therefore, determination of intracellular Ca^2+^ levels is employed to check for activation of GPR40 and GPR120. Activation of ERK1/2 has been confirmed as one of the downstream signaling cascades of GPR40-Gq and GPR120-Gq proteins signaling. We examined whether PAME increased the phosphorylation of ERK1/2 through activation of GPR40 and GPR120. As shown in (Figure 3(c)), cotreatment with PAME (50 *μ*M) and GW1100 or AH7614 together abolished the PAME-enhanced phosphorylation of ERK1/2. We further investigated the ERK1/2 upstream molecules. As shown in (Figure 3(d)), cotreatment with PAME (50 *μ*M) and U73122 (2 *μ*M), a PLC inhibitor, or BAPTA-AM (0.5 *μ*M), a Ca^2+^ chelator, abolished the PAME-induced phosphorylation of ERK1/2. The concentration of GW1100, AH7614, U73122, or BAPTA-AM used in this study did not affect the levels of p-ERK1/2 in rAD-MSCs after induction of adipogenic differentiation for 12 days (Supplement 1(a)). We also examined the intracellular Ca^2+^ levels in the PAME-treated rAD-MSCs by flow cytometric analysis using Fluo-3, an intracellular Ca^2+^ indicator. As shown in (Figures 4(a) and 4(b)), treatment with PAME (50 *μ*M) significantly increased the level of Fluo-3, and this effect was abolished by cotreatment with GW1100, AH7614, U73122, or BAPTA-AM. Moreover, cotreatment with PAME (50 *μ*M) and U73122, BAPTA-AM, or U0126 together also significantly reduced the PAME-enhanced adipogenic differentiation by Oil Red O staining, an indicator for adipogenic differentiation, in rAD-MSCs (Figures 5(a) and 5(b)). The PAME-increased mRNA levels of *Pparγ* (Figure 5(c)) and *Gpd1* (Figure 5(d)) and protein levels of PPAR*γ* were abolished in the cells cotreated with PAME and GW1100, AH7614, U73122, U0126, or BAPTA-AM (Figure 5(e)). The concentrations of these blockers used in this study did not affect protein levels of PPAR*γ* in rAD-MSCs after induction of adipogenic differentiation for 12 days (supplement 1(b)).

## 4. Discussion

PAME, a fatty acid ester of palmitic acid in mammalian cells, represents an endogenous naturally occurring FAME [34]. It has been reported that endogenous PAME is released from superior cervical ganglion [35], retinal [36], and perivascular adipose tissue [17]. Liu et al. showed that PAME was synthesized through PA methylation via the AdoMet-dependent catechol-O-methyltransferase catalytic pathway in adipocytes [37]. Recently, we detected PAME in the rat bone marrow [25]. Lee et al. reported that the culture medium of differentiated adipocytes of NIH 3T3 and 3T3-L1 cells contained a significant concentration of PAME [17]. PAME has been shown to inhibit phagocytosis [38, 39], fibrotic effects [18–20], inflammation [21, 22], and oxidative stress [40]; to induce vasodilation [17, 35, 36, 41, 42]; and to prevent nonalcoholic steatohepatitis [43]. In stem cells, PAME is able to induce cell cycle arrest at the G_2_/M phase in hBM-MSCs [25]. However, the molecular mechanisms underlying PAME-induced stem cell differentiation are still unclear. In the present study, we demonstrated that PAME activated the GPR40/120/PLC/ERK signaling pathway, leading to the enhancement of adipogenic differentiation in rAD-MSCs.

p53, also known as a tumour suppressor protein, can regulate cell cycle and promote differentiation of human embryonic stem cells and 3T3-L1 cells [44–48]. We previously showed that PAME was able to induce cell cycle arrest at the G_2_/M phase in hBM-MSCs via the p53/p21 pathway [25]. p53 can regulate the MAPK signaling pathways [49] and lead to accelerated differentiation of PC12 cells mediated by activation of the MAPK cascade [50]. In the present study, we demonstrated that PAME activated the PLC/ERK signaling pathway, leading to the enhancement of adipogenic differentiation in rAD-MSCs. It has been demonstrated that activation of the MEK/ERK signaling promotes adipogenesis by enhancing *Pparγ* and *C/ebpα* gene expression during the differentiation of 3T3-L1 preadipocytes [51]. ERK1 and ERK2 are involved in recruitment and maturation of hBM-MSCs induced to adipocyte differentiation [52, 53].

Calcium performs the physiological functions in cells by activating various intracellular signaling pathways. It is involved in several cell functions including apoptosis, exocytosis, and gene activation during the differentiation and/or proliferation processes. It has been demonstrated that increasing calcium in the early stages of differentiation inhibits human adipocyte differentiation, whereas increasing calcium in the late stage promotes human adipocyte differentiation and increases expression of PPAR*γ*, an adipogenic differentiation transcription factor [54]. In the present study, we demonstrated that PAME significantly increased the intracellular calcium levels, and cotreatment with BAPTA-AM, a Ca^2+^ chelator, abolished the PAME-enhanced adipogenic differentiation of rAD-MSCs and decreased the levels of *Pparγ* and *Gpd1* mRNA. It has been demonstrated that exogenous calcium treatment enhanced the adipogenic differentiation of human umbilical cord blood-derived mesenchymal stem cells via negatively regulating the Wnt5a/*β*-catenin signaling pathway [55]. In the present study, we demonstrated that PAME activated the PLC/ERK signaling pathway, and cotreatment with U73122 (a PLC inhibitor) or U0126 (an ERK inhibitor) abolished the PAME-enhanced adipogenic differentiation of rAD-MSCs and decreased the levels of *Pparγ* and *Gpd1* mRNA. Interaction between ERK and Wnt/*β*-catenin pathway can regulate proliferation and differentiation in embryonic stem cells [56, 57]. It has been demonstrated that stimulating the MEK-ERK pathway results in the activation of the Wnt/*β*-catenin pathway, leading to the differentiation of human embryonic stem cell towards the endoderm lineage [58]. Tian et al. showed that blockade of the ERK1/2 activity inactivates the Wnt/*β*-catenin signaling in the BM-MSCs and suggested a novel SPRY4-ERK1/2-Wnt/*β*-catenin regulatory loop, which plays a key role in cell adipogenic determination and in balancing of bone structure and homeostasis of bone marrow microenvironment [59].

GPR40/120 are receptors for both saturated and unsaturated fatty acids. It has been reported that a carboxylic group of the FFAs is required for GPR40 receptor activation. If the carboxylic group of FFAs is replaced by the methyl ester group, then the GPR40 receptor cannot be activated [60]. However, the relationship between the carboxylic group and GPR40 receptor activation has not been confirmed. Some studies showed that replacement of the carboxylic group with other different functional groups still can activate the GPR40/120 receptors [31, 61, 62]. In the present study, we demonstrated that PAME, which does not have carboxylic group, can still enhance adipogenic differentiation through activating GPR40/120. The *α*-linolenic acid has been shown to stimulate the release of glucagon-like peptide-1 in cultured STC-1 intestinal endocrine cells and in mice and rats [63, 64]. However, *α*-linolenic acid is an agonist of both GPR120 and GPR40, which are both expressed in the intestine, and GPR40 has also been shown to mediate the glucagon-like peptide-1 release [65]. Although GPR40 and GPR120 share only 10% homology between their amino acid sequences [60, 63], the binding activity of FAs to GPR120 and GPR40 is similar. Many of the natural agonists, such as EPA, DHA, and *α*-linolenic acid, activate both receptors, making the difficulty to distinguish which of these two receptors contribute a specific biologic process [66–68].

In the present study, we demonstrated that PAME can promote MSC adipogeneic differentiation. MSCs play an important role in adipogenesis, which can lead to obesity [69]. It has been reported that adipose tissue secretes numerous adipokines and FFAs, thereby promoting MSC differentiation into adipocytes. Obese patients have significantly elevated PA levels in the blood [70]. A recent study reported that PAME is biosynthesized from PA via catechol-O-methyltransferase catalysis [37]. Moreover, a study has suggested that catechol-O-methyltransferase may participate in the development of obesity [71]. These findings suggest that more adipose tissue will synthesize and release more PAME, which in turn causes more MSCs to differentiate into adipocytes, forming a vicious circle.

## 5. Conclusions

**Figure 6:**
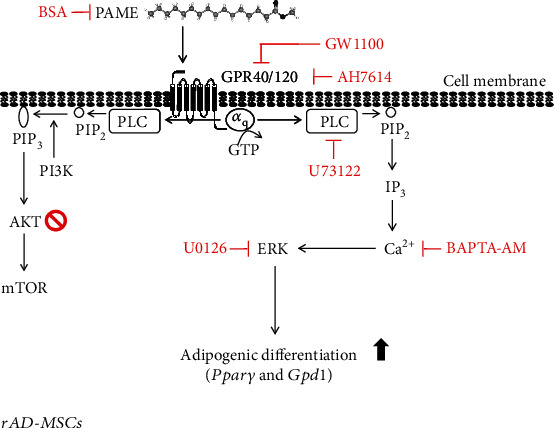
Schematic representation of the PAME-enhanced adipogenic differentiation in rAD-MSCs. PAME stimulated the GPR40 and GPR120, subsequently activating PLC, which in turn caused IP_3_ generation, thereby leading to ERK1/2 activation through increases of intracellular Ca^2+^ levels, and eventually increased the expression of adipogenic differentiation genes, *Pparγ* and *Gpd1*.

The experimental findings presented in this paper introduce our basic observation that PAME can induce adipogenic differentiation through activating the GPR40/120-mediated signaling pathway. We propose a model of the molecular mechanism underlying PAME-enhanced adipogenic differentiation in rAD-MSCs as shown in Figure 6. Although in vivo studies of adipocyte-derived PAME promoting the adipogenic differentiation of MSC and leading to obesity are still ongoing, the findings from the present in vitro studies suggest that inhibition of the PAME activities may be a new direction for consideration in animal models of obesity to increase the possibility of future clinical applications.

## Data Availability

All materials are available by the corresponding author.

## Supplementary Materials

Supplementary Materials Supplement 1: treatment with GW1100 (5 *μ*M), AH7614 (5 *μ*M), U73122 (2 *μ*M), or BAPTA-AM (0.5 *μ*M) in adipogenic induction medium for 12 days did not affect the activity of ERK1/2 (a) and the level of PPAR*γ* (b) in rAD-MSCs. The top panel shows Western blot analysis; the lower panel shows a graph of quantitation of these data adjusted with their own total protein (a) or *β*-actin (b). Values present the means of fold of corresponding control ± s.e.mean (*n* = 5). Treatment PAME (50 *μ*M) with 0.1% DMSO (Veh: vehicle), an antagonist solvent, in adipogenic induction medium for 12 days. The activity of ERK1/2 and the level of PPAR*γ* were not significantly different between the AIM+PAME and the AIM+PAME+Veh in rAD-MSCs (c).
