# Supplementary material for: Palmitic Acid Methyl Ester Enhances Adipogenic Differentiation in Rat Adipose Tissue-Derived Mesenchymal Stem Cells through a G Protein-Coupled Receptor-Mediated Pathway
Source: Stem Cells Int. 2021 Oct 5;2021:9938649. doi: 10.1155/2021/9938649 (PMC8510814; doi:10.1155/2021/9938649)
Supplement: Supplementary Materials — Supplement 1: treatment with GW1100 (5 μM), AH7614 (5 μM), U73122 (2 μM), or BAPTA-AM (0.5 μM) in adipogenic induction medium for 12 days did not affect the activity of ERK1/2 (a) and the level of PPARγ (b) in rAD-MSCs. The top panel shows Western blot analysis; the lower panel shows a graph of quantitation of these data adjusted with their own total protein (a) or β-actin (b). Values present the means of fold of corresponding control ± s.e.mean (n = 5). Treatment PAME (50 μM) with 0.1% DMSO (Veh: vehicle), an antagonist solvent, in adipogenic induction medium for 12 days. The activity of ERK1/2 and the level of PPARγ were not significantly different between the AIM+PAME and the AIM+PAME+Veh in rAD-MSCs (c). [file 9938649.f1.docx]

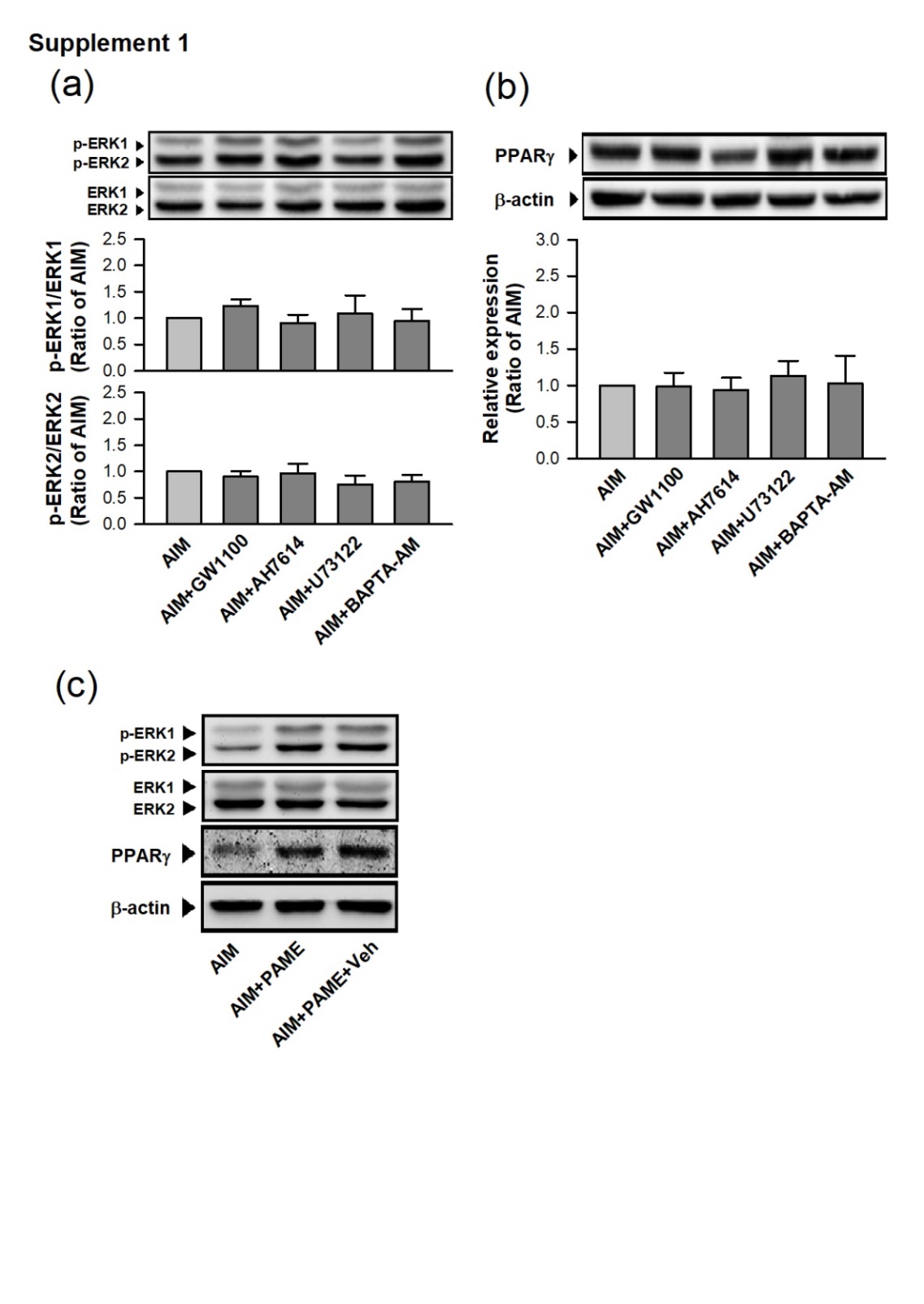
Supplement 1. Treatment with GW1100 (5 μM), AH7614 (5 μM), U73122 (2 μM), or BAPTA-AM (0.5 μM) in adipogenic induction medium for 12 days did not affect the activity of ERK1/2 (a) and the level of PPARγ (b) in rAD-MSCs. Top panel shows Western blot analysis; lower panel shows a graph of quantitation of these data adjusted with their own total protein (a) or β-actin (b). Values present the means of fold of corresponding control ± s.e.mean. (n = 5). Treatment PAME (50 μM) with 0.1% DMSO (Veh: vehicle), an antagonist solvent, in adipogenic induction medium for 12 days. The activity of ERK1/2 and the level of PPARγ were not significantly different between the AIM+PAME and the AIM+PAME+Veh in rAD-MSCs (c).
